# Healthcare worker-based opportunistic screening for familial hypercholesterolemia in a low-resource setting

**DOI:** 10.1371/journal.pone.0269605

**Published:** 2022-06-09

**Authors:** Sonali Sharma, Ashish Khudiwal, Sonal Bhardwaj, Hemant Chaturvedi, Rajeev Gupta

**Affiliations:** 1 Department of Biochemistry, RUHS College of Medical Sciences, Rajasthan University of Health Sciences, Jaipur, India; 2 Department of Preventive Cardiology, Eternal Heart Care Centre & Research Institute, Jaipur, India; 3 Academic Research Development Unit, Rajasthan University of Health Sciences, Jaipur, India; University of Alberta, CANADA

## Abstract

**Background & objective:**

Heterozygous familial hypercholesterolemia (FHeH) is important risk factor for premature coronary artery disease (CAD). Strategies for its diagnosis and prevalence have not been well studied in India. We performed healthcare worker-based opportunistic screening to assess feasibility for determining its prevalence.

**Methods:**

A healthcare worker was trained in use of Dutch Lipid Clinic Network (DLCN) criteria for diagnosis of FHeH. Successive eligible individuals (n = 3000 of 3450 screened) presenting to biochemistry laboratories of two hospitals for blood lipid measurements were evaluated for FHeH. Cascade screening or genetic studies were not performed. Descriptive statistics are reported.

**Results:**

We included 2549 participants (men 1870, women 679) not on statin therapy. Health worker screened 25–30 individuals/day in 6–10 minutes each. The mean age was 46.2±11y. Variables of DLCN criteria were more in women vs men: family history 51.1 vs 35.6%, past CAD 48.2 vs 20.1%, arcus cornealis 1.1 vs 0.3%, tendon xanthoma 0.3 vs 0.1%, and LDL cholesterol 190–249 mg/dl in 8.5 vs 2.4%, 250–329 mg/dl in 0.7 vs 0% and ≥330 mg/dl in 0.3 vs 0% (p<0.01). Definite FHeH (DLCN score >8) was in 15 (0.59%, frequency 1:170) and probable FHeH (score 6–8) in 87 (3.4%, frequency 1:29). The prevalence was significantly greater in women, age <50y and in those with hypertension, diabetes and known CAD.

**Conclusions:**

Healthcare worker-led opportunistic screening for diagnosis of FHeH using DLCN criteria is feasible in low-resource settings. The results show significant prevalence of clinically detected definite and probable FHeH in the population studied.

## Introduction

Multiple epidemiological, genome-wide and candidate-gene association, Mendelian randomization, mechanistic and experimental studies and randomized controlled clinical trials have confirmed importance of raised low-density lipoprotein (LDL) cholesterol as causal risk factor for coronary artery disease (CAD) [1, 2]. More than half of the raised LDL cholesterol is due to genetic factors and studies have identified that mutations in LDLR, APOB and PCSK9 genes are important [3]. Two types of genetic or familial hypercholesterolemia (FH) mutations have been described, (i) familial homozygous hypercholesterolemia (FHoH) and, (ii) familial heterozygous hypercholesterolemia (FHeH) [4]. FHoH is a rare Mendelian dominant disorder with prevalence of 1-2/500,000 while FHeH is a more common with prevalence of 1/200-250. Both present as premature CAD [5, 6]. It has been estimated that 5% of acute coronary syndromes in patients less than 60 years and about 20% at age less than 45 years are due to FHeH [7]. The worldwide prevalence of FH is based on studies conducted in European, North American, Australian and East Asian populations [3–7]. The Global Burden of Diseases (GBD) study reported high incidence of hypercholesterolemia related mortality in most Asian countries [8]. Non Communicable Disease Risk Factor Collaboration (NCDRisC) has reported that currently the highest burden of raised non-HDL cholesterol is in South-East and South Asian countries [9]. Studies have reported high prevalence of FH in China and other Asian countries [10]. The prevalence of FH has not been well studied in India [11]. A multisite population-based study reported prevalence of severe hypercholesterolemia (LDL cholesterol ≥190mg/dl) in 1:300 individuals [12]. A study among school children in Delhi also reported prevalence of 1:300 [13]. On the other hand hospital based case-series [14] and cascade screening have reported greater prevalence [15, 16].

Guidelines recommend population-based screening for early identification and management of FHeH to prevent and delay onset of clinical CAD [17]. Screening strategies include primary care clinic-based population screening, general population-based screening with HFeH diagnosed using computerized predictive algorithm, cascade screening of families of patients with known premature CAD or severe hypercholesterolemia and opportunistic screening at primary or secondary care clinics [18, 19]. Genetic studies have been advised to confirm its diagnosis [3]. Many of these strategies are expensive and may not be suitable for low-resource settings in lower-middle and low-income countries, where premature CAD burden is the highest [8]. We performed a health-worker led and hospital clinical laboratory based opportunistic screening to determine feasibility and prevalence of FHeH using the Dutch Lipid Clinic Network (DLCN) criteria [20].

## Methods

The study was conducted in out-patient departments of a government and a non-government private hospital in Rajasthan, India. The study was approved by institutional ethics committees of both the institutions and written informed consent was obtained from all the study participants.

### Health worker training

A graduate non-physician health worker was identified and trained for a month prior to initiation of the study. A mentorship approach was adopted and training was performed through a structured training programme as in a previous study at our centre [21]. This individual was trained to inquire details of history of premature CAD in the participants’ family using the DLCN questionnaire and identification of arcus on corneal examination. Tendon xanthomas are rare occurrence and therefore only theoretical training using photographs was performed. The training was initially provided in the preventive cardiology department and supplemented by teaching by experienced clinicians (HC, RG). Initially photographs were used for identification of the physical stigmata (corneal arcus, tendon xanthomas) and later by real-life cases. For the first 10% cases, the participant was evaluated by the health worker as well as by the clinicians at both the hospitals. Subsequently, the clinicians were available on-site to provide support to the health worker. Details of anthropometric data (height, weight, blood pressure) were also recorded. We performed comparison of the health worker evaluations with clinician’s response in the first 200 cases following the training. The results showed good concordance with coefficient of variation of <5%. Cascade screening of family members of affected participants was not performed due to resource constraints.

### Screening

All consecutive individuals in age-group 18–70 years reporting for fasting lipid profile at the hospital biochemistry laboratories were screened over a 6-month period from October 2019 to March 2020 (n = 3450) to achieve the target of 3000 participants. Participants with acute or chronic infectious diseases, secondary hypercholesterolemia such as nephrotic syndrome, acute or chronic kidney disease, acute or chronic liver disease or hypothyroidism were excluded. Only those participants who were fasting for 8–10 hours prior to blood sample collection for biochemical assessments-total cholesterol, LDL cholesterol, high density lipoprotein (HDL) cholesterol and triglycerides- were included. Fasting blood sample was collected using vacuum blood-collection tubes containing clot activator for lipid profile estimation. Serum cholesterol were measured using enzymatic methods with a fully-automated clinical chemistry analyzer (Beckman Coulter AU680) at both the sites. Total cholesterol was measured using CHOD-PAP method, triglycerides using GPO-PAP method and LDL cholesterol and HDL cholesterol by direct enzymatic methods. Both the laboratories follow stringent external quality control program to ensure validity and reliability. Data on lipid profile (total cholesterol, directly measured LDL and HDL cholesterol, and triglycerides) were recorded. We did not perform genetic studies to confirm diagnosis of familial hypercholesterolemia.

The eligible participants were classified into definite FHeH, probable FHeH and possible FHeH according to the Dutch Lipid Clinics Network (DLCN) diagnostic criteria [20]. These criteria create a numerical score based on personal and family history of premature CAD, physical examination (arcus, xanthomata), LDL cholesterol levels and genetic mutations and provide a diagnosis of definite, probable or possible FHeH. The data were interpreted as definite FHeH when score >8 points, probable FHeH with score 6–8 points, possible FHeH with score 3–5 points and no FHeH when the score was < 3. Individuals diagnosed with FH were referred to clinicians for confirmation and provide consultation for risk management. No genetic screening data are available.

### Statistical analyses

Demographic, clinical and biochemical characteristics of participants have been summarised using descriptive statistics. Categorical variables are presented as percent and continuous variables as mean ± 1 standard deviation (SD). To identify significance of inter-group differences we used Chi-square test for categorical variables and t-test for continuous variables. P values <0.05 are considered significant.

## Results

We screened 3450 individuals presenting to the laboratory for estimation of lipid profile over the 6-month period and included 3000 eligible and consenting adults. 25–30 individuals were screened daily (mean 28/day) over this period. The average time taken for evaluation of each participant was 6–10 minutes (mean 8.6 minutes/person). We excluded 451 participants who were on statin therapy. Data of 2549 individuals (men 1870, women 679) have been analysed for presence of FHeH. Mean age of participants was 46.2±11.1 years (men 47.4±11.0, women 42.8±10.7, p<0.01). Prevalence of known CAD was in 173 (6.8%) (men 6.3%, women 8.3%); family history of CAD in 1143 (44.8%) (men 48.3%, women 35.4%, p<0.001); smoking/tobacco in 973 (38.2%) (men 50.0%, women 5.6%, p<0.001); hypertension in 610 (23.9%) (men 26.2%, women 17.7%, p<0.01); diabetes in 268 (10.5%) (men 9.5%, women 13.1%, p<0.001); and obesity (body mass index, BMI ≥25 kg/m^2^) in 1105 (43.5%)(men 38.3%, women 57.1%, p<0.001). Mean levels of cholesterol lipoproteins and triglycerides in the study cohort were-total cholesterol 210.0 ± 98.4 mg/dl, LDL cholesterol 120.5 ± 60.1 mg/dl, HDL cholesterol 47.4 ± 14.9 mg/dl, non-HDL cholesterol 160.6 ± 98.0 mg/dl and triglycerides 157.1 ± 67.4 mg/dl. In men vs women, no significant differences were observed for total cholesterol (210.1 ± 102.4 vs 210.0 ± 86.7 mg/dl), non-HDL cholesterol (163.8 ± 102.7 vs 159.4 ± 87.6 mg/dl) and triglycerides (158.2 ± 67.0 vs 154.0 ± 68.4 mg/dl) (p>0.05). LDL cholesterol (116.6 ± 45.7 vs 131.3 ± 87.5 mg/dl) and HDL cholesterol (46.4 ± 13.8 vs 50.4 ± 17.1 mg/dl) were significantly higher in women (p<0.01). In men vs women, categories of higher total cholesterol and triglycerides were not significantly different, while prevalence of raised LDL cholesterol >160 mg/dl (men 19.5%, women 27.7%) and >190 mg/dl (men 2.3%, women 9.4%) were significantly more in women (p<0.01) (Table 1).

**Table 1:** Cholesterol lipoproteins and triglycerides in the study cohort.

Variable	Total(n = 2549)	Men(n = 1870)	Women(n = 679)	χ^2^test (p-value)
**Total cholesterol, mg/dl**				
** <170**	970 (38.1)	722 (38.6)	248 (36.5)	7.598 (0.022)
** 170–200**	580 (22.8)	398 (21.3)	182 (26.8)	
** >200–240**	407 (16.0)	306 (16.4)	101 (14.9)	
** >240–270**	183 (7.2)	146(7.8)	37 (5.5)	
** >270–300**	126 (4.9)	99 (5.3)	27 (4.0)	
** >300**	283 (11.1)	199 (10.6)	84 (12.4)	
**LDL cholesterol, mg/dl**				
** <70**	529 (20.8)	432 (23.1)	97 (14.3)	28.251 (<0.001)
** 70–100**	504 (19.8)	342 (18.3)	161 (23.7)	
** >100–130**	410 (16.1)	288 (15.4)	122 (18.0)	
** >130–160**	553 (21.7)	443 (23.7)	111 (16.3)	
** >160–190**	445 (17.5)	321 (17.2)	124 (18.3)	
** >190–220**	89 (3.5)	36 (1.9)	53 (7.8)	
** >220**	19 (0.8)	8 (0.43)	11 (1.6)	
**HDL cholesterol, mg/dl**				
** <30**	153 (6.0)	119 (6.4)	34 (5.0)	19.233(<0.001)
** 30–44**	1136 (44.6)	894 (47.8)	242 (35.6)	
** 45–60**	949 (37.2)	669(35.8)	280(41.2)	
** 60+**	311 (12.2)	188 (10.1)	123 (18.1)	
**Non-HDL cholesterol, mg/dl**				
** <100**	614 (24.1)	474 (25.3)	140 (20.6)	14.985(<0.001)
** 100–130**	472 (18.5)	318 (17.0)	154 (22.7)	
** >130–160**	518 (20.3)	359 (19.2)	159 (23.4)	
** >160–190**	350 (13.7)	267(14.3)	83 (12.2)	
** >190–220**	171 (6.7)	138 (7.4)	33 (4.9)	
** >220–250**	107(4.2)	83(4.4)	24(3.5)	
** >250**	317(12.4)	231(12.4)	86(12.7)	
**Triglycerides, mg/dl**				
** <150**	1061 (41.6)	738 (39.5)	323 (47.6)	13.074(0.001)
** 150–250**	1305 (51.2)	993 (53.1)	312(45.9)	
** >250–350**	130 (5.1)	98 (5.2)	32(4.7)	
** >350–450**	40 (1.6)	32 (1.7)	8(1.2)	
** >450**	13 (0.5)	9 (0.5)	4(0.6)	

Numbers in parentheses are percent. LDL low density lipoprotein; HDL high density lipoproteins.

**Fig 1:**
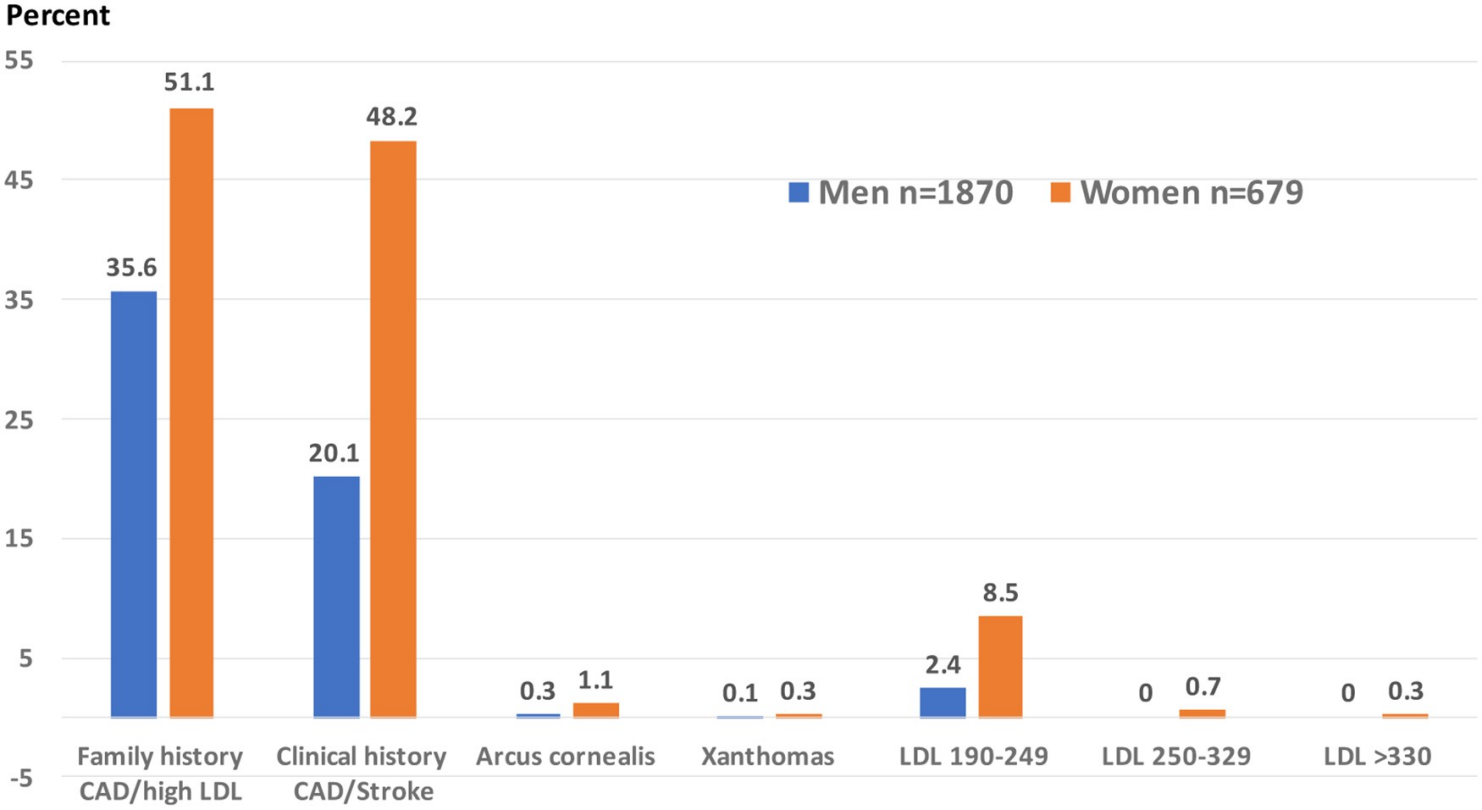
Components of Dutch Lipid Clinic Network FH diagnostic criteria in men and women in the study cohort.

Various components of Dutch Lipid Clinics Network criteria for FHeH in the study population are shown in Fig 1. Family history of CAD and/or high cholesterol was in 1012 (39.7%), clinical history of premature CAD or stroke in 712 (27.9%), tendon xanthomas in 3 (<0.1%) and arcus cornealis in 13 (0.5%). All these clinical variables and high LDL cholesterol groups were significantly more in women as compared to men (Fig 1). We also determined the prevalence and frequency of definite and probable FHeH in the study population using DLCN criteria (Table 2). Definite FHeH (DLCN score >8) was in 15 (0.59%) with frequency of 1:170; and probable FHeH (DLCN score 6–8) in 87 (3.4%), frequency 1:29. Prevalence of definite and probable FHeH was significantly greater in women, participants <50 years and in those with hypertension, diabetes and known CAD.

**Table 2:** Prevalence of definite and probable familial hypercholesterolemia in sub-groups.

Variable	Number	Definite FH		Probable FH	
		Prevalence	Frequency	Prevalence	Frequency
**Total cohort**	2549	15 (0.59)	1:170	87 (3.41)	1:29
**Men**	1870	6 (0.32)	1:311	38 (2.03)	1:49
**Women**	679	9 (1.33)	1:75	49 (7.2)	1:14
**Age** **≤50 years**	1464	12(0.82)	1:122	78 (5.33)	1:19
**Age >50 years**	1085	3 (0.28)	1:362	9 (0.83)	1:121
**CAD Family history**	1143	13(1.1)	1:88	39(3.4)	1:29
**Smoking/tobacco**	973	10(1.0)	1:97	35(3.6)	1:28
**Hypertension**	610	13(2.1)	1:50	16(2.6)	1:38
**Diabetes**	268	4(1.5)	1:67	14(5.2)	1:19
**Obesity (BMI >25 kg/m** ^**2**^ **)**	1105	8(0.7)	1:138	18(1.6)	1:68
**Known CAD**	173	3(1.7)	1:58	13(7.5)	1:13

## Discussion

This study shows that healthcare worker based opportunistic screening for diagnosis of familial hypercholesterolemia using Dutch Lipid Clinics Network clinical diagnostic criteria (without genetic testing) is feasible in a low-resource setting in India, a lower middle income country. The results also show that this strategy has led to identification of significant prevalence of definite and probable HFeH and the data are similar to previously published studies among South Asians in the UK [22].

A WHO Consultation for early diagnosis and treatment of FH recommended multiple strategies for improved identification, treatment and long term follow-up [23]. These included identification of index cases with known or suspected FH, contacting relatives to find other persons needing testing and treatment for FH, creation of registry of FH patients and their physicians, and education of patients and physicians with regard to formal treatment [23]. Our study is focused on identification of index cases only. Referral to physicians was performed but creation of a registry and cascade screening were not done. Routine genetic testing was not recommended by the WHO Consultation [23].

Recent case-finding studies for FH use multiple strategies ranging from opportunistic screening of individuals in primary care, identification of FH using physician-led or computerized algorithms and cascade screening of families to identify at-risk individuals [18, 19]. Such screening strategies have also been performed in secondary care and specialist cardiac centers for identification of FH in patients with known CAD followed by cascade screening of families [3, 4, 13, 18, 19]. All these strategies are either physician focused or technology focused and may not be suitable for populations in lower-middle income countries such as in South and South East Asia, the global epicenters of hypercholesterolemia [9]. There is evidence that nonphysician health workers can render multiple aspects of care in communicable as well as non-communicable diseases [17, 21, 24, 25]. Population based evaluation for FH using large clinical databases has been performed in Scandinavian countries while large population-based epidemiological studies have been conducted in many European and North American countries [3, 5, 6]. In low and lower middle income countries use of non-physician health-worker led evaluation for CAD risk factors- hypertension, diabetes and others chronic conditions is a more practical method for identification of individuals with high cardiovascular risk [21, 25, 26]. The present study confirms usefulness of non-physician health worker for diagnosis of FH using opportunistic screening strategy and is an example of task redistribution [27]. Given the lack of physicians and excessive workload, especially in lower-middle and low- income countries, it would be worthwhile to consider training healthcare workers as a task -sharing approach to screen individuals for FH at the community level and further refer to the clinician for early intensive treatment. In our study the specific and detailed patient interaction by healthcare worker which lead to clinical decision making took about 6–10 minutes per patient and could be a cost-effective strategy, although we did not perform a formal cost-effectiveness analysis.

Beheshti et al performed a meta-analysis including data of 11 million population-based individuals in 17 countries [6]. The pooled global prevalence of FHeH involving 44 population-based studies and random-effects modelling was 0.32% (95% CI 0.26–0.39%) with frequency of 1:313. The prevalence was higher in studies that included participants with known CAD (6.7%, 95% CI 4.9–8.7%) and premature IHD (7.2%, 95% CI 4.6–10.8%). The review also reported that there was no difference in FH prevalence when either genetic or clinical criteria were used, our findings could be considered as representative accordingly. Substantial heterogeneity has been reported in prevalence of FH in various countries and among ethnic subgroups. A study in UK reported that FH likelihood was the highest in South Asian and lowest in Afro-Caribbean ethnic groups in a study of 770,000 primary care patients [22]. A study in European Mediterranean countries involving 2.5 million individuals in primary care reported FH in 0.58%, frequency 1:172 [28], while in Denmark prevalence of genetically determined FH was reported in 0.73%, frequency 1:137 [29]. In Japan, Teramoto et al reported lower prevalence of definite or suspected FH of 0.8% in 187,781 individuals [30]. A review based on PubMed database search up to March 2020 concluded that prevalence of HeFH could be more than 2-fold greater than previously reported [31]. In India, a multisite population-based study at 11 urban locations (n = 5350) reported prevalence of severe hypercholesterolemia (LDL cholesterol ≥190mg/dl) in 1.4% (frequency 1:357) [12], while a hospital-based study (n = 67347) reported it in 2.2% (frequency 1:209) [14]. Another hospital-based study in CAD patients in India reported higher HFeH prevalence (definite 4%, probable 11%) [15]. The results of our study with a much smaller sample and using health-worker based opportunistic screening shows prevalence of HFeH in 1:176 and is similar to many of these studies.

The study has multiple limitations and strengths. The study was performed at a secondary care government hospital and tertiary care non-government hospital and this could have led to a selection bias. Primary care or population-based sampling is desirable for identification of prevalence. Secondly, use of non-physician health worker for evaluation for HFeH using the DLCN criteria may have led to missed subtle clinical findings of arcus cornealis or tendon xanthomas. To overcome this, we utilised a physician at the government hospital to validate the results in the first 200 participants. Thirdly, laboratories at 2 different sites were used for biochemical studies and to overcome this we used similar technology and reagents at both the sites with external validation. Fourthly, hospital based opportunistic screening is not the best method to determine prevalence of FHeH, but the primary aim of the present study was to demonstrate feasibility of using a non-physician health worker for diagnosis while prevalence of FHeH is a co-primary outcome. Fifthly, we did not perform genetic analysis of enrolled participants as recommended by some international agencies and guidelines [20, 32], due to high costs. Sixthly, we did not perform cascade screening of individuals identified as having FHeH, nor did we perform repeated biochemical evaluation for confirmation of FHeH. And finally, the study lacked follow up of diagnosed FH patients for long term management. The strength of the study is demonstration of feasibility of a simple and effective strategy for FHeH screening using limited infrastructure and resources.

In conclusion, the study demonstrates feasibility of hospital laboratory based non-physician health worker-led strategy for diagnosis of familial hypercholesterolemia with user friendly DLCN criteria sans genetic analyses. The evidence of high prevalence of FH in the study demonstrates that leveraging the existing infrastructure and task redistribution strategy to address health system barriers decentralizes the diagnosis of FH. This healthcare delivery model could be easily translated at multiple sites across the globe for identification of FHeH, the most important CAD risk factor [1, 2]. Early identification of pathologically raised LDL cholesterol and its management by aggressive lifestyle interventions (exercise, healthy diet) and statins can lead to prevention of premature CAD [33]. This is all the more important as CAD is the most important cause of premature CAD among South Asians [34].
